# Association of Hepatitis B Virus Mutations of A1846T and C1913A/G With Acute-on-Chronic Liver Failure Development From Different Underlying Chronic Liver Diseases

**DOI:** 10.5812/hepatmon.12445

**Published:** 2013-09-01

**Authors:** Aimin Zhang, Zhihong Wan, Shaoli You, Hongling Liu, Bing Zhu, Jing Chen, Yihui Rong, Hong Zang, Chen Li, Huifen Wang, Shaojie Xin

**Affiliations:** 1 Liver Failure Treatment and Research Center, Beijing 302 Hospital, Beijing, China

**Keywords:** Liver Failure, Hepatitis B Virus, Mutation, Risk Factors

## Abstract

**Background:**

As most HBV-related acute-on-chronic liver failure (ACLF) have concurrent cirrhosis, it is important to clarify the association of viral factors with ACLF with or without cirrhosis.

**Objectives:**

The aim of this study was to analyze the association of HBV genotypes and mutations with ACLF development underlying different chronic liver diseases.

**Patients and Methods:**

Eighty-seven ACLF patients including 29 patients with chronic hepatitis (ACLF-CHB) and 58 patients with liver cirrhosis (ACLF-LC) were enrolled. Age and sex matched patients with chronic hepatitis (CHB) and liver cirrhosis (LC) were enrolled as controls. The genotypes and mutations at HBV basic core promoter (BCP), precore (PC), and partial C regions were determined by nested PCR and direct sequencing.

**Results:**

Our results revealed significantly higher incidences (P < 0.05) of genotype B with C1913A/G or A1846T in patients with ACLF-CHB than those with CHB; genotype C with C1913A/G or A1846T in patients with ACLF-CHB and ACLF-LC than those with CHB and LC, respectively. Multivariable analysis indicated that A1846T and C1913A/G mutations were independent factors for ACLF (OR = 2.86 and 5.93, respectively), suggesting an association between the mutations and development of ACLF. In addition, there were no significant differences in mutations at T1753V, A1762T, G1764A, G1896A, and G1899A which were found between either CHB and ACLF-CHB or LC and ACLF-LC patients， suggesting no associations of these mutations with ACLF development.

**Conclusions:**

Our findings suggest that CHB or LC patients infected with HBV A1846T and C1913A/G mutants are more susceptible to develop ACLF.

## 1. Background

Acute-on-chronic liver failure (ACLF) encompasses patients with previously well-compensated liver disease in whom an acute decompensation of liver function occurs due to a precipitating event. The following definition of ACLF was recommended by the Asian Pacific Association for the Study of the Liver (APASL) in 2009: Acute hepatic insult manifesting as jaundice and coagulopathy, complicated within 4 weeks by ascites and/or encephalopathy in a patient with previously diagnosed or undiagnosed chronic liver disease (1). In China, HBV-infected ACLF patients account for more than 80% of ACLF cases owing to a high incidence of chronic HBV infection (2,3).

Although detail mechanisms for liver failure caused by hepatitis B virus (HBV) infection are unclear, previous studies suggested that variations in the viral genome, such as genotypes or mutations, might play a role in liver disease progression (4, 5, 6, 7). The mutations in the precore region (PC), such as G1896A, generated a stop codon and halt translation of HBeAg. Failure to produce HBeAg might help the HBV to evade immune detection. In addition, the mutation in the basic core promoter (BCP), such as double mutation A1762T/G1764A, has been found to enhance HBV replication in vitro. These mutations in BCP/PC region were reported to be associated with ACLF (8, 9). Also, genotypes B, T1753V, and G1899A were more frequently found in ACLF patients than CHB patients (10, 11, 12). However, previous studies mainly focused on the associations between those hot spot mutations in BCP/PC regions and HBV-related ACLF. It is unclear whether other mutations in these regions were associated with the progression of liver failure.

HBV-related ACLF is an acute event on an underlying liver disease, which can develop from patients with chronic hepatitis or cirrhosis. ACLF patients with cirrhosis usually have severe clinical and pathological symptoms in comparison to patients with chronic hepatitis, suggesting poor prognoses in ACLF patients underlying cirrhosis (13, 14). It is important to categorize ACLF patients into cirrhotic and noncirrhotic to determine their clinical features, treatment, and prognosis. In most of the Asian countries, cirrhosis constitutes about 70% of underlying liver diseases of HBV-induced ACLF (15, 16). In comparison with CHB patients, liver cirrhosis (LC) patients have more frequent mutations in BCP/PC region such as A1762T/G1764A, T1753V, and/or G1896A (15, 17, 18). The effect of these mutants on the development of ACLF might be masked by persisting liver cirrhosis. Unfortunately, most of previous studies did not categorize ACLF patients into cirrhotic and noncirrhotic, and viral factors with ACLF might be related to cirrhosis rather than ACLF. Therefore, it would be of interest to clarify BCP/PC mutants in ACLF patients with different persistent underlying chronic liver diseases (CHB or LC).

## 2. Objectives

The aim of this study was to analyze HBV genotypes and mutations in HBV-related ACLF patients, and identify the viral factors associated with ACLF development from different underlying chronic liver diseases (CHB or LC).

## 3. Patients and Methods

### 3.1. Patients

Sample size was calculated based on the formula: n = (U_α_+U_β_)2/(P1-P0)2, and expected power was 0.9. From February 2009 to April 2010, the patients who hospitalized in the Liver Failure Treatment and Research Center of Beijing 302 Hospital in Beijing, China were included. Among them, eighty-seven patients met the diagnostic criteria of ACLF and enrolled in the study. ACLF was diagnosed according to the criteria recommended by the Chinese Society of Infectious Disease and the Chinese Society of Hepatology (2). ACLF patients met the following criteria: recent development of increasing jaundice (Total bilirubin 171.0 µmol/L or rapid increase to17.1 µmol/L/day) and decreasing Prothrombin activity (< 40%), with a recent development of complications. Two groups of age and sex matched controls, including 52 patients with CHB and 51 patients with LC were included during the same study period. The clinical diagnosis of CHB and LC conformed to the 2000 Xi’an criteria (19).

For all patients, no special antiviral treatment (such as interferon, lamivudine, or adefovir) and no immunosuppressive medication were applied at the time of the study. There was no evidence of concurrent Hepatitis C virus (HCV), Hepatitis D virus (HDV), Hepatitis G virus (HGV), or Human Immunodeficiency Virus (HIV) infection. Furthermore, there was no evidence of autoimmune liver disease or hepatocellular carcinoma.

The protocol had been approved by the Ethical Committee of Beijing 302 Hospital on Feb.1, 2008 (code: 2008005D) and conformed to the ethical guidelines of the 1975 Declaration of Helsinki. Written informed consent was obtained from each patient before entering the study protocol.

### 3.2. Clinical Parameters

The biochemical parameters were routinely performed in the Central Clinical Laboratory of Beijing 302 Hospital. The presence of HBsAg and HBeAg was determined using commercial assay kits (Kewei Diagnostic Ltd., China). The HBV DNA was quantified using a quantitative fluorescent PCR kit (Fuxing Clone Co., China) with a detection limit of 500 copies/ml.

### 3.3. Typing of HBV Genotypes

The genotypes were determined using nested PCR based on the surface gene (nt 256-796) and direct sequencing as described previously (20).

### 3.4. Amplification and Direct Sequencing of HBV BCP/PC/C Regions

**Figure 1.**
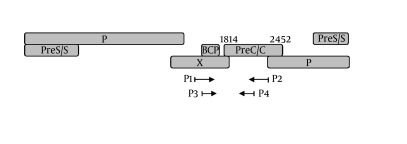
Schema of the PCR established to detect the mutations at basic core promoter (BCP), precore (PC) and partial core of HBV (BCP/PC/C). BCP region includes nt 1751-1769, Pre C/C region includes nt 1814-2452.

Viral DNA was extracted and subjected to a nested PCR as reported before ( 21 ). A DNA segment composing of the BCP, precore, and partial C regions was amplified by PCR. The Schema of HBV genome and the position of PCR primers were shown in Figure 1. The primers P1 (5’-TCG CAT GGA GAC CAC CGT GA-3’, nt 1604-1623) and P2 (5’-ATA GCT TGC CTG AGT GC-3’, nt 2076-2060) were used for the first round; primers P3 (5’-CAT AAG AGG ACT CTT GGA CT-3’, nt 1653-1672) and P4 (5’-GGA AAG AAG TCA GAA GGC-3’ nt 1974-1957) for the second round. Amplified PCR products were purified by Qiaquick spin columns (Qiagen Inc., Hilden, Germany). Sequencing was performed with an ABI PRISM 3730xl DNA Analyzer by using BigDye terminator v3.1. (Applied Biosystems, Foster City, CA). Analysis and assembly of sequencing data were performed with the Vector NTI Suite 9.0 software (Informax, Frederick, MD). 

### 3.5. Statistical Analysis

The Vector NTI Suite 9.0 software was used to align nucleotide sequences, and data processing was performed with Stata v.11. Continuous variables were expressed as mean ± standard deviation (SD). Analysis was determined using a 2-tailed Student’s t-test. Parameters with non-normal distribution were expressed as median (min-max) and compared by Mann-Whitney U-test. Categorical data was compared with an X2 test. Multivariate analyses with logistic regression were used to determine independent factors. A P value less than 0.05 was considered as statistically significant.

## 4. Results

### 4.1. Clinical Features and Virological Characteristics of ACLF Patients With Underlying CHB or LC 

Of the 87 ACLF patients, 29 (33.3%) had CHB (ACLF-CHB) and 58 (66.7%) had compensated LC (ACLF-LC). Comparison of clinical and virological characteristics between ACLF-CHB and ACLF-LC patients were summarized in Table 1. In comparison with ACFL-CHB patients, a similar level (P > 0.05) of serum total bilirubin and prothrombin activity was observed in ACLF-LC patients. The frequency of HBeAg expression and viral load showed no significant difference between these two groups. HBV genotype B and C were detected in 19 and 68 patients of ACLF, respectively. The ratio of genotype B to C was 24.4% in ACLF-CHB patients and 20.7% in ACLF-LC patients (P = 0.714). The incidence of BCP/PC/C mutants was 100% in ACLF-LC patients and 89.7% in ACLF-CHB patients, respectively, suggesting significantly higher BCP/PC/C mutations occurred in ACLF patients with underlying cirrhosis (P < 0.05). Furthermore, ACLF-LC patients had significantly higher frequency (P < 0.05) of BCP mutations at T1753V, A1762T, and G1764A than ACLF-CHB patients. In addition, since BCP/PC mutations of T1754G, T1758C, C1766T, T1768A, G1862T, and T1858C were observed only in 7, 5, 7, 4, 1, and 4 of ACLF patients, respectively, these mutations were not included in further analysis. 

**Table 1.** Clinical and Virological Characteristics of HBV-Related ACLF: Comparison Between Patients with Chronic Hepatitis and Liver Cirrhosis

	Total ACLF (n = 87)	ACLF-CHB (n = 29)	ACLF-LC (n = 58)	P value
**Age, y**	46.1 ± 11.9	43.4 ± 10.5	47.4 ± 12.5	0.142
**Gender (male/female)**	75/12	27/2	48/10	0.187
**Total Bilirubin (µmol/L)**	336.2 (170.8 - 659.1)	330.2 (172.6 - 659.1)	338.4 (175.5 - 627.8)	0.989
**Alanine aminotransferase (U/L)**	91.5 (10 - 2131)	93 (10 - 1932)	91.5 (20 - 2131)	0.898
**Prothrombin activity, No. (%)**	26 (5 - 40)	21.5 (5 - 38)	27 (5 - 40)	0.569
**HBV DNA (logIU/mL)**	3.97 ± 1.76	3.86 ± 1.57	4.02 ± 1.85	0.691
**HBeAg (positive/negative)**	37/50	11/18	26/32	0.829
**Genotype (B/C)**	19/68	7/22	12/46	0.714
**BCP/PC/C mutations (Total) , No. (%)**	84 (96.5)	26 (89.7)	58 (100)	0.013
**T1753C/A/G, No. (%)**	34 (39.1)	7 (24.1)	27 (46.6)	0.043
**A1762T, No. (%)**	61 (70.9)	14 (48.3)	47 (81.0)	0.002
**G1764A, No. (%)**	60 (69.0)	14 (48.3)	46 (79.3)	0.003
**A1762T/G1764A, No. (%)**	57 (65.5)	13 (44.8)	44 (75.9)	0.004
**A1846T, No. (%)**	49 (56.3)	19 (65.5)	33 (56.9)	0.879
**G1896A, No. (%)**	46 (52.9)	15 (51.7)	31 (53.5)	0.879
**G1899A, No. (%)**	15 (17.2)	4 (13.8)	11 (19.0)	0.547
**C1913A/G, No. (%)**	42 (48.3)	13 (44.8)	29 (50)	0.649

### 4.2. Comparison of BCP/PC/C Mutations of HBV in ACLF-CHB and CHB Patients, ACLF-LC and LC Patients, Respectively

ACLF is an acute event which occurs in the liver with underlying chronic liver disease. Both CHB and LC can develop to ACLF. We next sought to compare the BCP/PC/C mutations between ACLF-CHB and CHB patients, ACLF-LC and LC patients, respectively. The results were summarized in Table 2. The BCP/PC/C mutations were found in all patients with LC and ACLF-LC. In comparison with CHB patients, a slight increase of BCP/PC/C mutations was observed in ACLF-CHB patients (89.7% vs. 76.9%, P = 0.157). The prevalence of the A1846T mutation in HBV PC region was 65.5%, and 56.9% in patients with ACLF-CHB and ACLF-LC, respectively, which was significantly higher than those in CHB, and LC patients (P < 0.05). Meanwhile the C1913A/G mutation in HBV C region was significantly increased in patients with ACLF-CHB, and ACLF-LC than those with CHB and LC, respectively (P < 0.001). However there were no significant differences in previous reported hotspot mutations (T1753V, A1762T, G1764T, G1896A, and G1899A) between either CHB and ACLF-CHB patients or LC and ACLF-LC patients.

**Table 2.** Virological Characteristics Among Age and Sex Matched Patients with CHB and ACLF-CHB, LC and ACLF- LC, Respectively

	CHB (n = 52)	ACLF-CHB (n = 29)	P value ^a^	LC (n = 51)	ACLF-LC（n = 58）	P value ^b^
**Age, y**	42.3 ± 11.6	43.4 ± 10.5	0.673	46.2 ± 11.1	47.4 ± 12.5	0.599
**Gender (male/female)**	42/10	27/2	0.134	39/12	48/10	0.414
**HBV DNA (logIU/mL)**	4.63 ± 2.51	3.86 ± 1.57	0.139	4.69 ± 2.04	4.02 ± 1.85	0.075
**Genotype (B/C)**	16/36	7/22	0.526	8/43	12/46	0.501
**HBeAg (positive/negative)**	32/20	11/18	0.058	19/32	26/32	0.423
**BCP/PC/C mutations (Total)**	40 (76.9)	26 (89.7)	0.157	51 (100)	58 (100)	1.000
**T1753C/A/G, No. (%)**	11 (21.2)	7 (24.1)	0.104	22 (43.1)	27 (46.6)	0.721
**A1762T, No. (%)**	25 (48.1)	14 (48.3)	0.986	44 (86.3)	47 (81.0)	0.462
**G1764A, No. (%)**	26 (50.0)	14 (48.3)	0.882	44 (86.3)	46 (79.3)	0.339
**A1762T/G1764A, No. (%)**	25 (48.1)	13 (44.8)	0.779	43 (84.3)	44 (75.9)	0.273
**A1846T, No. (%)**	15 (28.8)	19 (65.5)	0.001	17 (33.3)	33 (56.9)	0.014
**G1896A, No. (%)**	23 (44.2)	15 (51.7)	0.517	28 (54.9)	31 (53.5)	0.879
**G1899A, No. (%)**	2 (3.9)	4 (13.8)	0.101	11 (21.6)	11 (19.0)	0.735
**C1913A/G**	5 (9.6%)	13 (44.8%)	<0.001	9 (17.7%)	29 (50%)	<0.001

^a^ACLF-CHB vs. CHB

^b^ACLF-LC vs. LC

### 4.3. Analysis of BCP/PC/C Mutations in ACLF-CHB and CHB Patients, ACLF-LC and LC Patients According to HBV Genotype 

**Figure 2.**
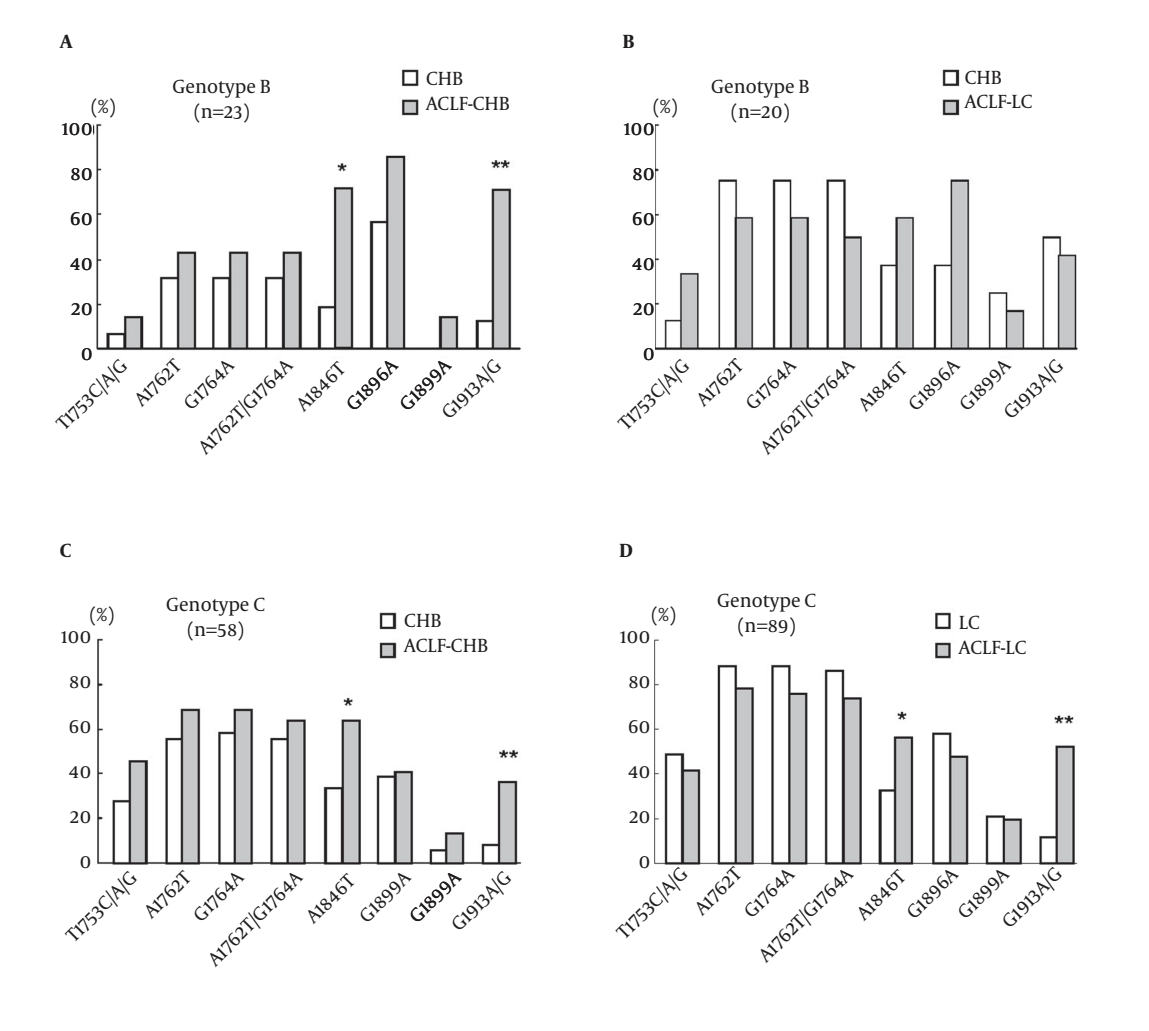
Comparison of BCP/PC/C mutation incidences in different illness categories according to HBV genotypes. (A) Comparison of mutations in HBV genotype B between patients with CHB (n = 16) and ACLF-CHB (n = 7). (B) Comparison of mutations in HBV genotype B between patients with LC (n = 8) and ACLF-LC (n = 12). (C) Comparison of mutations in HBV genotype C between patients with CHB (n = 36) and ACLF-CHB (n = 22). (D) Comparison of mutations in HBV genotype C between patients with LC (n = 43) and ACLF-LC (n = 46). * P < 0.05; ** P < 0.01

To study the frequency of BCP/PC/C mutations in different HBV genotypes, the rates of BCP/PC/C mutations in genotype B and C viruses were compared. As shown in Figure 2, HBV genotype B and C were detected in 43, and 147 of all patients, respectively. In genotype B infected patients, a significantly higher prevalence of A1846T and C1913A/G mutations was observed in the HBV isolates of ACLF-CHB patients in comparison with those of CHB patients (71.4% vs. 18.8%, P < 0.05; 71.4% vs. 12.5%, P < 0.005). While there were no significant differences in mutations observed in ACLF-LC patients in comparison with LC patients. In genotype C infection, a statistically higher prevalence (P < 0.05) in the occurrence of mutations was observed at A1846T and C1913A/G in patients with ACLF-CHB and ACLF-LC than those with CHB and LC, respectively. 

### 4.4. Possible Factors Associated with the Development of Acute-on-Chronic Liver Failure

Multiple logistic regression analyses were performed to determine the independent contribution of clinical features and viral factors in development of ACLF. HBV genotypes, HBeAg status, and frequencies of the BCP/PC/C mutations (T1753V, A1762T, G1764T, A1846T, G1899A, and C1913A/G) were included in the study. The results were summarized in Table 3. The presence of A1846T in PC region (OR: 2.86, 95% CI: 1.58−5.19, P < 0.01) and the C1913A/G in the C region (OR: 5.93; 95% CI: 2.260–6.894; P < 0.001) were independently associated with the development of ACLF, suggesting that A1846T and C1913A/G were independent risk factors for ACLF. However, HBV genotypes, HBeAg status, and other mutations did not show a significant association with ACLF development in multivariate analyses. 

**Table 3.** Possible Factors Associated with the Development of HBV Associated Acute-on-Chronic Liver Failure

Factors	Odds Ratio	95% Confidence Interval (CI)	P value
**C1913A/G**	5.93	2.94 − 11.99	< 0.001
**A1846T**	2.86	1.58 − 5.19	0.001
**T1753C/A/G**	1.36	0.75 − 2.47	0.312
**G1899A**	1.44	0.64 − 3.23	0.372
**A1762T**	1.55	0.51 − 1.83	0.430
**G1764A**	1.21	0.64 − 2.26	0.560
**HBeAg**	1.33	0.75 − 2.35	0.336
**genotype**	1.09	0.55 − 2.15	0.810

## 5. Discussion

In this study, the mutations of HBV BCP, precore and partial core region in ACLF patients were investigated by categorizing the patients into cirrhotic and noncirrhotic. We showed that the HBV precore mutation of A1846T and core mutation of C1913A/G (substitution at the fifth amino acid: proline to threonine or alanine) were associated with HBV-related ACLF development.

ACLF is an acute event underlying different liver diseases including HBV associated compensated liver cirrhosis (LC) or chronic hepatitis (CHB). The different underlying liver diseases may affect the ACLF development. In comparison with CHB patients, the HBV isolates in LC patients had higher frequencies of the mutations in BCP/PC region, which included A1762T/G1764T, T1753V, and/or G1896A (22, 23). We found that ACLF patients underlying LC had a high prevalence of the BCP mutations than those underlying CHB, suggesting that these BCP mutations were associated with liver cirrhosis. When ACLF patients were categorized into ACLF-CHB and ACLF-LC based on their underlying liver diseases, a significantly higher prevalence of the A1846T, and C1913A/G mutations were observed compared to respective CHB or LC patients. This observation is different from previous studies in which the mutations of T1753V, A1762T, G1764T, G1896A, and G1899A were found to be more prevalent in ACLF patients. These results support our hypothesis that those previous reported BCP/PC mutations (T1753V, A1762T, G1764T, G1896A, and G1899A) might be associated with liver cirrhosis, but not ACLF development. The effect of these mutants on the development of ACLF might be masked by persisting liver cirrhosis. A similar finding was reported recently by Chu et al (24). They demonstrated that HBV BCP mutants are associated with progression to cirrhosis rather than HCC in chronic HBV infection.

HBV genotype is a factor which may influence the course of liver disease, especially in Asia where genotypes B and C are prevalent (23, 25). Previous reports had indicated that genotype C had a higher frequency of BCP mutations, which is thought to be a reason for the relationship between genotype C and severe liver disease (26, 27). In the present study, we did not find any difference in the ratio of genotype B and C between patients with ACLF-CHB and CHB or ACLF-LC and LC. A1846T or C1913A/G mutation in both genotypes B and C was associated with ACLF from CHB. Genotype C with A1846T or C1913A/G mutation was associated with ACLF development from LC. Furthermore, multivariate analysis demonstrated that A1846T and A/G1913 were independent factors for ACLF, with odds ratios of 2.86 and 5.93, respectively. As the numbers of patients with ACLF underlying CHB were relatively small, further studies of a larger series of patients are needed to confirm these observations.

**Figure 3.**
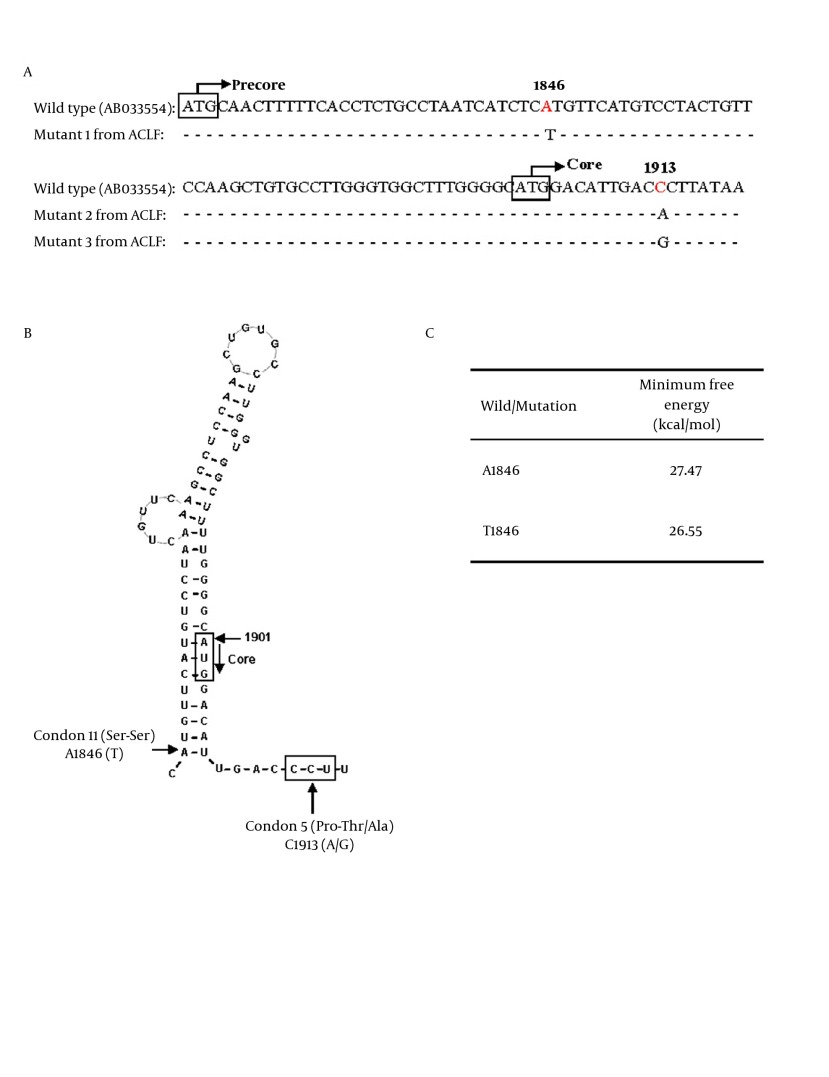
Possible influences of A1846T and C1913A/G mutations on core protein expression. (A) Partial sequences around HBV nt 1846 and 1913. The HBV wild type sequence was shown in the first line for comparison. A1846T comprised 65.5%, and 56.9% of ACLF-CHB and ACLF-LC patients. C1913A/G comprised 44.8%, and 50% of ACLF-CHB and ACLF-LC patients. Mutants 1 to 3 represented the HBV sequences from 3 of ACLF patients. The white box showed the initial codons of precore/core protein. (B) Proposed secondary structure of the HBV ε signal with A1846T and C1913A/G mutations. A1846 is at the mouth of RNA secondary structure. The C1913A/G mutation causes a substitution of the fifth amino acid in core protein from proline to threonine or alanine. (C) Stability of the ε signal evaluated with minimum free energy which was calculated using RNAdraw software

HBV ε signal which forms a secondary structure of viral pregenomic RNA is essential for initiating encapsidation of pregenomic RNA ( 28 ). Positions of A1846T and C1913A/G mutations compared to HBV wild type were shown in Figure 3A. The nt.1846 is at the mouth of the stem-loop structure (Figure 3B). Previous studies showed that the A1846T was found in patients with fulminant hepatitis and ACLF ( 6 , 12 , 29 ). The mechanism is unknown. Minimum free energy keeps the stability of ε signal. The lower energy value indicates higher stability of the structure. Our results showed that the ε signal with A1846T mutation had lower value of minimum free energy, suggesting a higher stability than wild type HBV (Figure 3C). The stabilized ε signal has an advantage for pregenomic RNA encapsidation and is considered to heighten replication efficiency of HBV ( 28 ). The HBV core antigen represents an important target for immune mediated viral clearance by inducing B cell, T helper cell, and cytotoxic T lymphocyte (CTL) responses ( 30 ). Important recognition sites of the core protein are represented by the amino acid sequences 18-27, 88-96 and 141-151 for the CTL epitopes and amino acid sequences 1-20, 28-47, 50- 69, 72-105 and 108-165 for T helper epitopes ( 30 ). Nucleotide 1912-1914 of HBV encodes the fifth amino acid (Proline) in the core protein, which is located within an HLA II-restricting helper T cell of HBcAg. The C1913A/G mutation causes a substitution of the fifth amino acid of the core protein from proline to threonine or alanine (Figure 3A, B). The amino acid substitution may have been selected to evade immune recognition ( 31 , 32 ). Since proline is usually found at the turns of protein secondary structure, loss of a proline may cause a structural change of the core protein. Thus, HBV of A1846T and A/G1913 mutation may promote ACLF development through either enhancing HBV replication or core protein expression. 

In conclusion, HBV precore mutation of A1846T and the fifth amino acid substitution in the core protein due to C1913A/G were found more frequently in ACLF patients than in their respective CHB or LC patients. This finding may give an insight into the mechanism of ACLF underlying different liver diseases, and may be useful to predict the development of ACLF. Further in vitro experiments and mechanistic studies are needed to support this issue.
